# The Mediating Role of Self-Esteem in the Relationship Between Loneliness and Phubbing: Evidence from a Cross-Sectional Study

**DOI:** 10.3390/jcm14238588

**Published:** 2025-12-04

**Authors:** Joanna Furmańska, Magdalena Dworakowska, Maja Gębarowska, Aleksandra Grzanka, Małgorzata Szcześniak

**Affiliations:** Institute of Psychology, University of Szczecin, 71-017 Szczecin, Polandmagda.dworakowska1@wp.pl (M.D.); maja.gebarowska@wp.pl (M.G.); grzanka.ola05@gmail.com (A.G.)

**Keywords:** loneliness, phubbing, self-esteem, mediating factor

## Abstract

**Background/Objectives:** In today’s reality, the mobile phone accompanies people in almost every area of life. Technological progress offers a range of conveniences, facilities, and opportunities. At the same time, researchers observe new phenomena such as *phubbing*, which is defined as ignoring others in favor of one’s smartphone and is increasingly being perceived as a normative behavior. **Methods:** The study was conducted using an online survey. A total of 201 adults aged between 18 and 75 participated. The research employed a proprietary questionnaire designed to collect data on phone and social media use, as well as the Revised UCLA Loneliness Scale (R-UCLA), the Generic Scale of Phubbing (GSP), and the Self-Esteem Scale (SES). **Results:** The results showed a positive relationship between loneliness and phubbing, and a negative relationship between loneliness and self-esteem. Additionally, a negative relationship was found between self-esteem and phubbing behavior. In line with the main objective of the study, it was demonstrated that self-esteem acts as a mediating factor in the relationship between loneliness and phubbing behavior. **Conclusions:** Individuals experiencing loneliness may have lower self-esteem, which in turn may lead them to engage in phubbing behavior more frequently. Identifying factors related to phubbing behavior helps expand knowledge about this new, yet increasingly common phenomenon, which carries psychosocial consequences. At the same time, the topic highlights the need for further research to deepen our understanding of the phenomena of loneliness and phubbing.

## 1. Introduction

In the digital era, the use of mobile phones has become a widespread phenomenon, accompanying people in almost every aspect of daily life. Smartphones are used for communication, education, work, and entertainment, among other purposes. Undoubtedly, technological progress and digital devices offer a wide range of opportunities and conveniences. At the same time, researchers have observed new phenomena, such as increasing reliance on and attachment to smartphones and social media [1,2,3,4], fear of missing out (FoMO) [5,6,7], and phubbing [8]. As a widespread social phenomenon, phubbing is increasingly being perceived as normative behavior [9,10]. It seems that researchers’ interest in both the new opportunities and the potential challenges and consequences associated with these behaviors is important not only for academic discourse but also for providing practical implications for mental health professionals.

### 1.1. Phubbing as a Common Phenomenon

Phubbing represents a relatively new area of research interest [8,11,12]. In their review of qualitative and mixed-method studies on phubbing, Deschamps et al. [13] highlighted the diverse ways researchers define this phenomenon—for instance, as simply using a smartphone in a social context [14] or as the act of ignoring physically present individuals [15]. In simple terms, the phenomenon refers to behaviors in which a person looks at and uses their phone while simultaneously engaging in conversation with someone else [8]. Consequently, phubbing can be considered a form of both social exclusion and neglect of interpersonal relationships [16], as displaying phubbing behaviors may be perceived as dismissive and communicatively avoidant [17]. Moreover, according to the theory of the norm of social inclusion [18,19], people expect attention during communication processes [20].

Vanden Abeele [21], in describing the social consequences of phubbing, identified three sociocognitive mechanisms involved in this behavior: (a) expectancy violations, (b) ostracism, and (c) attentional conflict. Deschamps et al. [13], based on their literature review, also identified negative consequences of phubbing, which they categorized into those related to: (a) feelings and perceptions, (b) mental health, (c) lifestyle, (d) social life and relationships, and (e) work and academic settings. Moreover, their review highlighted an additional point: although all the studies they examined reported negative consequences of phubbing, some also discussed positive aspects associated with smartphone use among individuals who engage in phubbing. Specifically, smartphones have been cited as facilitating access to information [15], providing entertainment [15,22], enabling communication [23], and even reducing loneliness [24] or strengthening a sense of belonging [25].

Due to the ubiquity of smartphones and their frequent use—even during social interactions—the practice of phubbing has become increasingly common. It can occur anywhere and at any time, across various social gatherings [26], and is therefore linked to multiple types of interpersonal relationships, including romantic relationships [27], family dynamics [28], workplace interactions [29], and friendships [30].

Furthermore, Chotpitayasunondh and Douglas [11,31] point to a self-perpetuating cycle in which phubbing behavior predicts the extent to which individuals themselves become victims of being “phubbed.” In addition, phubbing has been associated with the unintended consequences of modernization [32], meaning that although people use technological innovations for numerous activities, they often overlook the social conditions arising from technological progress [33]. Karadağ et al. [8] emphasize that phubbing is a multidimensional phenomenon, involving mental health–related factors such as addictions to (a) mobile phones, (b) the Internet, (c) social media, and (d) gaming.

Regarding the causes of phubbing, Arenz et al. [34], based on a meta-analytic review of 79 studies and 526 effect sizes, identified ten higher-level predictor categories of phubbing behavior: sociodemographics, personality, technology-related norms and experiences, technical equipment, (smart)phone and Internet use, problematic use, well-being, psychopathology, resilience, and risk factors. They also noted that the strongest predictors were problematic use patterns. Previous research indicates a negative association between social media addiction and relationship satisfaction [35], as well as with phubbing behaviors [36,37,38].

Al-Saggaf and O’Donnell [39] observed that although technological addictions, boredom, fear of missing out, and lack of self-control all predict phubbing behavior, smartphone use during face-to-face interactions may also be linked to a tendency toward multitasking and the pursuit of immediate needs or gratification.

In terms of personality traits, neuroticism and conscientiousness have been identified as predictors of phubbing [40]. With respect to communication style, avoiding, angry, belittling, and manipulative interpersonal communication styles positively predict phubbing, whereas a dominant communication style predicts it negatively [41].

Furthermore, loneliness has been identified as a potential factor intensifying phubbing behavior [8], which is particularly important given its relevance as a contemporary public health concern.

### 1.2. Loneliness as a Public Health Problem

Human beings are inherently social, and social connections constitute an important component of a good life [42] and overall well-being [43]. However, there is growing discussion today about the “opposite end” of this spectrum—loneliness—which has become a widespread experience [44] and even a public health concern [45,46]. Approximately one-third of people in industrialized countries report experiencing loneliness [46].

Culture has also been shown to influence loneliness [47], with individualism identified as one of its predictors [48]. Loneliness is understood as an aversive feeling arising from the absence of meaningful relationships that an individual needs [49]. It is therefore a subjective experience resulting from the discrepancy between desired and actual social relationships [50]. Consequently, loneliness differs from social isolation, which refers to an objective lack of social contacts within one’s environment [51,52].

Previous empirical findings have linked loneliness to reduced well-being [53], poorer mental health [54], including depressive disorders [55,56] and anxiety disorders, as well as to physical health issues [57]. Recent studies have also shown that individuals with higher levels of loneliness are more likely to engage in phubbing behaviors. This tendency has been interpreted as a coping mechanism for dealing with feelings of isolation and withdrawal, promoting the search for alternative forms of interaction and, consequently, more frequent phone use [32,58,59].

Furthermore, research has demonstrated that loneliness—and social relationships more broadly—is associated with self-esteem. According to sociometer theory [60,61], individuals who experience interpersonal rejection tend to have lower self-esteem. Meanwhile, the reflected appraisals theory [62,63] emphasizes the role of others’ evaluations in shaping one’s self-perception—meaning that the self is experienced “through the eyes of others” [64].

Cacioppo et al. [55] found that higher levels of loneliness predict lower self-esteem, and Hawkley and Cacioppo [65] similarly demonstrated that loneliness reduces self-esteem. Research consistently reports a negative relationship between loneliness and self-esteem [66,67], and this association appears to be reciprocal: individuals who feel lonely tend to evaluate themselves more negatively, while low self-esteem may, in turn, contribute to heightened feelings of loneliness. In line with this reasoning, the present study aims to further examine the role of self-esteem as a potential mediating factor in the relationship between loneliness and phubbing behavior.

### 1.3. Self-Esteem as a Mediator

In the context of social relationships, self-esteem is a crucial element of social adaptation. It plays an important role in interpersonal communication and mediates social interactions between individuals and their environment [68]. According to the self-broadcasting perspective [69], individuals hold beliefs about how they are perceived by others and send signals that allow others to infer their level of self-esteem through behavioral expressions of confidence or the lack thereof [70]. The specific relationship behaviors perspective further suggests that self-esteem influences behavior, which in turn affects the quality of social relationships [61].

Given this, it is essential to analyze the role of self-esteem in the context of loneliness and phubbing. Research indicates that loneliness, which is associated with a lack of social bonds and belonging, leads to decreased self-esteem [66,71,72]. Previous empirical findings also suggest that self-esteem mediates the relationship between social media use and loneliness [73], as well as between depressive temperament and phubbing [74].

This overview clearly highlights the importance of the issue and supports examining self-esteem as a key factor closely related to two increasingly prevalent psychosocial phenomena: loneliness and phubbing behavior.

### 1.4. Knowledge Gap and the Purpose of Current Study

Human beings are social creatures, and social connections constitute an important aspect of a good life [42] and overall well-being [43]. Although phubbing behaviors are becoming increasingly common, further research is needed to deepen our understanding of this phenomenon. The topic is inherently interdisciplinary, attracting the attention of researchers in psychology, sociology, and related fields.

Studies have shown that phubbing is associated with behavioral addictions related to online activities, including smartphone and social media use [75,76,77], as well as with various types of interpersonal relationships [27,29]. Additionally, researchers have examined the negative impact of smartphones on well-being and mental health [69,70], and decreases in life satisfaction have also been reported [23].

Negative emotional responses—such as annoyance or sadness—have been observed among individuals who are being phubbed [15]. These findings underscore the need for a deeper understanding of the phubbing phenomenon, particularly since phubbing behaviors are often interpreted through the lens of social norms or contextual factors [13]. This suggests that in certain situations or social groups, using a smartphone during face-to-face interaction may be more acceptable than in others.

With regard to the motives for smartphone use among individuals who engage in phubbing, Deschamps et al. [13] note that beyond educational, professional, or communication-related reasons, smartphone use is often driven by feelings of boredom, stress, awkwardness, discomfort, fear of missing out, or loneliness. Particular attention has been drawn to loneliness, which has become an important public health concern. Although recent studies have identified a relationship between loneliness and phubbing behavior, we argue that, beyond this direct association, there may be mediating factors that play a significant role. To the best of our knowledge, no research to date has examined self-esteem as a potential mediator between loneliness and phubbing.

This focus is particularly justified for several reasons. First, self-esteem plays a key role in social interactions [78]. According to sociometer theory, self-esteem reflects perceived relational value, meaning that the quality of social relationships can influence an individual’s self-esteem [79]. Conversely, the self-broadcasting perspective proposes that self-esteem shapes social relationships [69,80]. Thus, self-esteem is embedded within the social environment and functions as an internal indicator of how individuals assess their relational value to others [61]. Consequently, a feedback loop can be assumed in which self-esteem may influence loneliness [81,82], but may also result from it [83].

Moreover, self-esteem is important in the context of susceptibility to negative, technology-related phenomena. Research highlights a negative correlation between experiencing phubbing and self-esteem [12] and indicates that higher self-esteem is associated with more adaptive coping mechanisms [84,85], which may be relevant when confronting a “phubber.” Analyzing self-esteem as a mediator allows for a deeper understanding of the psychological mechanisms through which loneliness and phubbing are connected and how they jointly influence overall well-being. Thus, self-esteem may not only be an outcome but also a driving force behind the negative consequences of social and digital interactions. In this way, it forms part of a complex, reciprocal cause-and-effect mechanism—a feedback loop. As an adaptive trait, self-esteem is closely associated with psychological well-being [86].

An additional objective of this study was to examine whether levels of loneliness, phubbing, and self-esteem differ across age groups—specifically between younger and older adults—as well as between men and women. Given that most individuals under the age of 30 have not yet made key adulthood-related life decisions, particularly concerning marriage and parenthood, it is reasonable to expect differences between younger adults and those over 30 in terms of interpersonal experiences, perceptions of self-worth, and smartphone use. Similar differences may also occur between men and women.

To address these gaps, the first aim of the present study was to assess the direct relationship between loneliness and phubbing. Based on previous empirical findings, we hypothesized that loneliness would be associated with higher levels of phubbing behavior, as such behaviors may serve as compensatory mechanisms for insufficient social relationships and as coping strategies for loneliness.

A second aim was to examine self-esteem as a mediating factor in the relationship between loneliness and phubbing behavior. Loneliness has been associated with lower levels of self-esteem, whereas reduced self-esteem predicts higher levels of phubbing behavior. Thus, we sought not only to assess the direct relationship between loneliness and phubbing but also to address a gap in understanding the potential mediating mechanisms underlying this association in adults. We anticipate that our findings may have important implications for understanding the motivational underpinnings of phubbing behaviors.

In accordance with the proposed research model, the study aimed to answer the following questions: (1) Is there a relationship between loneliness, phubbing, and self-esteem?; (2) Is self-esteem related to phubbing?; (3) Do loneliness, phubbing, and self-esteem differ by age group (emerging adults vs. older adults) and by sex (men vs. women)?; (4) Does self-esteem mediate the relationship between loneliness and phubbing?

Based on these research questions and previous empirical findings, the following hypotheses were formulated:

**H1:**  *Loneliness (total, intimate others, social others, belonging and affiliation) correlates positively with phubbing (total, nomophobia, interpersonal conflict, self-isolation, problem acknowledgment).*

**H2:**  *Loneliness (total, intimate others, social others, belonging and affiliation) correlates negatively with self-esteem.*

**H3:**  *Self-esteem correlates negatively with phubbing (total, nomophobia, interpersonal conflict, self-isolation, problem acknowledgment).*

**H4:**  *Emerging adults display higher levels of loneliness and phubbing, as well as lower self-esteem, compared to adults over the age of 30.*

**H5:**  *Women differ from men in loneliness, phubbing, and self-esteem, showing lower levels of loneliness, phubbing, and self-esteem compared to men.*

**H6:**  *Self-esteem mediates the relationship between loneliness (total, intimate others, social others, belonging and affiliation) and phubbing (total, nomophobia, interpersonal conflict, self-isolation, problem acknowledgment).*

## 2. Materials and Methods

### 2.1. Participants and Procedure

A total of 201 individuals participated in the study, ranging in age from 18 to 75 years (*M* = 31.78, *SD* = 14.03). The sample consisted of 65.2% women and 34.8% men. Non-probability sampling methods (convenience and snowball sampling) were employed. Prior to participation, all respondents provided informed consent. To minimize missing data, the survey was designed so that participants could proceed to the next section only after completing each item, resulting in a complete dataset. Exclusion criteria included being under 18 years of age or not providing consent to participate.

### 2.2. The Revised UCLA Loneliness Scale (R-UCLA)

The R-UCLA scale, developed by Russell et al. [87] and adapted into Polish by Kwiatkowska et al. [88], is a self-report instrument designed to measure loneliness. It consists of 20 items grouped into three subscales. The first dimension, “intimate others,” refers to feelings of detachment, isolation, and a lack of intimate contact with significant others (e.g., “There is no one I can turn to”). The second dimension, “social others,” reflects a lack of connection with other people and limited availability of social contact (e.g., “I can find companionship when I want it”). The third dimension, “belonging and affiliation,” captures the sense of belonging to a social group (e.g., “I have a lot in common with the people around me”). Each item is rated on a 4-point Likert scale (1 = never, 4 = often). A total score can also be calculated. The reliability of the scale has been confirmed in multiple studies, with Cronbach’s alpha values ranging from 0.89 to 0.94. In the present study, reliability was also very good: total score—α = 0.91; “intimate others”—α = 0.85; “social others”—α = 0.85; and “belonging and affiliation”—α = 0.71.

### 2.3. The Generic Scale of Phubbing (GSP)

The GSP, developed by Chotpitayasunondh and Douglas [11] and translated into Polish by Cieślak et al. [89], is a self-report scale consisting of 15 items grouped into four factors: (1) “nomophobia”, reflecting fear of being separated from one’s phone; (2) “interpersonal conflict”, referring to conflicts between one’s phone use and interactions with others; (3) “self-isolation”, describing the use of a smartphone to withdraw from others and from social activities; (4) “problem acknowledgment”, indicating awareness of having a phubbing problem. The GSP also provides a composite score representing overall phubbing behavior, defined as ignoring direct social interactions in favor of smartphone use. Each item is rated on a 7-point Likert scale (1 = never, 7 = always). In the original version, the reliability of the scale was demonstrated with Cronbach’s alpha values ranging from 0.82 to 0.93. In the present study, reliability was also very good: total score—α = 0.88; “nomophobia”—α = 0.81; “interpersonal conflict”—α = 0.84; “self-isolation”—α = 0.82; and “problem acknowledgment”—α = 0.67.

### 2.4. The Self-Esteem Scale (SES)

The SES, developed by Rosenberg [90] and adapted into Polish by Łaguna et al. [91], is a brief scale consisting of 10 items forming a single dimension that reflects an individual’s overall attitude toward themselves. Each item is rated on a 4-point Likert scale (1 = strongly disagree, 4 = strongly agree). The reliability of the scale has been confirmed across different age groups, with Cronbach’s alpha values ranging from 0.81 to 0.83. In the present study, internal consistency was satisfactory (α = 0.88).

### 2.5. Statistical Analysis

Statistical analyses were conducted using IBM SPSS Statistics, version 25 (Predictive Solutions, Kraków, Poland). The distribution of variables was evaluated according to the criterion proposed by Tabachnick and Fidell [92], which states that skewness and kurtosis values within ±2 indicate an approximately normal distribution. As this criterion supported the assumption of approximate normality, Pearson’s *r* correlations were employed. Mediation analyses were performed using the PROCESS macro for SPSS developed by Hayes [93], applying the bootstrapping method with 5000 resamples and 95% confidence intervals (CIs).

An a priori power analysis was conducted using G*Power 3.1.9.4 with a bivariate normal correlation model [94] to determine the required sample size. A small effect size of 0.21, an alpha level of 0.05, and a desired statistical power of 0.90 were specified. The choice of this effect size was based on an extensive review of social psychology literature by Richard et al. [95], who estimated the average effect size across published findings to be 0.21. The analysis indicated that a minimum of 187 participants would be required.

## 3. Results

### 3.1. Descriptive Statistics

In accordance with the adopted research model, basic descriptive statistics were first calculated and are presented in Table 1.

### 3.2. Relationship Between Loneliness, Phubbing, and Self-Esteem

The results revealed a statistically significant positive correlation between loneliness and phubbing, as well as with most dimensions of both constructs, largely supporting Hypothesis 1. Only the belonging and affiliation dimension did not correlate significantly with either nomophobia or problem acknowledgment, and social others did not correlate significantly with nomophobia. Furthermore, a statistically significant negative relationship was found between loneliness (and all its dimensions) and self-esteem, supporting Hypothesis 2. In other words, individuals with higher levels of loneliness tend to exhibit lower levels of self-esteem. The present study also found a statistically significant negative relationship between self-esteem and phubbing behaviors (total score and all dimensions), which is consistent with Hypothesis 3. This indicates that lower levels of self-esteem are associated with more frequent phubbing behaviors. Table 2 presents these findings.

The analysis (Table 3) revealed statistically significant differences between emerging adults and older adults across most of the examined variables, largely supporting Hypothesis H4. Emerging adults reported higher overall levels of loneliness than older adults, a pattern also reflected in the subscales assessing intimate relationships and social connections. However, no significant differences emerged for the belonging and affiliation dimension. Emerging adults also scored significantly higher on phubbing and nomophobia, indicating a greater tendency toward problematic smartphone-related behaviors. In addition, they reported higher levels of interpersonal conflict and self-isolation, suggesting greater vulnerability to the negative relational consequences associated with excessive smartphone use. Higher scores on the problem acknowledgment subscale further indicate that emerging adults were more likely to recognize difficulties related to their smartphone use. In contrast, older adults reported significantly higher levels of self-esteem than emerging adults, consistent with previous findings on age-related differences in general self-worth.

The analysis (Table 4) showed that most of the examined variables did not differ significantly between women and men. Women scored slightly lower than men on loneliness, social connections, phubbing, self-isolation, problem acknowledgment, and self-esteem, whereas men scored slightly higher on belonging and affiliation and intimate relationships. The only statistically significant differences were observed for belonging and affiliation and for nomophobia, with men reporting higher scores on belonging and affiliation and women reporting higher levels of nomophobia. Overall, these results indicate that gender differences in this sample were limited. Therefore, Hypothesis H5 was not supported.

### 3.3. Mediating Analysis

In the subsequent analyses (Table 5), the main aim of the study was addressed—namely, to examine self-esteem as a potential mediator in the relationship between feelings of social isolation and smartphone-related neglect of social interactions (Hypothesis H6). Using mediation analyses based on Hayes’ [73] approach and the PROCESS macro for SPSS, the results indicated that loneliness, as the predictor, was associated with lower self-esteem, which in turn was linked to increased phubbing behaviors.

The mediation models were statistically significant, as confirmed by the bootstrapped confidence intervals (Table 5), with the exception of the model examining social others and interpersonal conflict with self-esteem as the mediator. These findings indicate that self-esteem played a significant role in the relationship between loneliness (and its dimensions) and phubbing (and its dimensions). More specifically, across nearly all outcomes, self-esteem emerged as an important mediator, consistently explaining how loneliness—whether conceptualized as intimate others, social others, or belonging and affiliation—is associated with more problematic technology-related behaviors, such as phubbing (including nomophobia, interpersonal conflict, self-isolation, and problem acknowledgment), through reduced levels of self-esteem.

## 4. Discussion

The aim of the study was to examine the direct relationship between loneliness and phubbing, as well as to identify self-esteem as a mediating factor in this relationship. All hypotheses proposed in the theoretical model were confirmed.

First, the results indicate a positive relationship between loneliness and phubbing (H1), which is consistent with previous empirical findings [58,59], suggesting that loneliness is a significant predictor of phubbing [32]. Smartphone use may serve as a compensatory response to a lack of social relationships and may function both as a coping mechanism and a means of seeking connection and belonging [96,97,98]. In other words, loneliness—as a psychological and social problem—may be associated with excessive mobile phone use [99] and with phubbing [59]. Individuals who engage in phubbing may inadvertently harm their interpersonal relationships and well-being, further increasing loneliness and reducing self-esteem [100]. In this context, excessive use of digital devices, or even technology addiction, may reinforce isolation [101], while individuals experiencing loneliness may be more prone to phubbing as a way to find virtual contact with others [97]. Taken together, these mechanisms demonstrate a reciprocal, self-perpetuating cycle that may intensify technology-related addictions [102].

The second hypothesis, predicting a negative relationship between loneliness and self-esteem (H2), was also confirmed—a finding consistent with earlier empirical evidence [65,67,81]. Lonely individuals may be more susceptible to social comparison, which, especially when unfavorable, can contribute to declines in self-worth [103]. Given the reciprocal nature of the relationship between loneliness and self-esteem, dissatisfaction with the quality of social relationships and the experience of loneliness may be associated with negative self-evaluations and lower self-esteem [66,71], while low self-esteem may hinder the formation of satisfying social relationships and deepen loneliness [61].

Engagement in social comparison has also been linked to lower self-esteem [104,105]. Individuals with lower self-esteem tend to exhibit higher levels of fear of missing out (FoMO) [106], and may engage in phubbing behaviors [107] as a coping strategy for feelings of insecurity and discomfort in social interactions [108]. The findings of the present study support this pattern (H3), highlighting the complexity of the underlying mechanisms.

In line with Hypothesis H4, emerging adults exhibited significantly higher levels of loneliness and phubbing, along with significantly lower self-esteem, compared to adults over the age of 30. These results align with both theoretical perspectives and empirical findings. For instance, according to the evolutionary theory of loneliness [81], younger individuals—whose brain regions responsible for cognitive control are still developing—may be particularly sensitive to social cues. As a result, they may be especially vulnerable to loneliness [109]. Regarding phubbing, Winkelmann and Geber [110] found that younger adults engaged in phubbing more frequently during interactions with peers compared to older adults. Additionally, a meta-analysis of longitudinal studies by Orth et al. [111] showed that self-esteem increases from childhood into the late twenties, continues to rise gradually through middle age, peaks around age 60, remains stable until about age 70, and then declines in very old age. These findings confirm that emerging adults tend to exhibit lower self-esteem than older adults, consistent with our results.

Hypothesis H5 was largely unsupported, except for two variables that showed significant sex differences. Men reported higher levels of belonging and affiliation, whereas women scored higher on nomophobia. The first result is somewhat unexpected, as many studies report higher loneliness among women. However, Barreto et al. [112], in a large-scale study involving 46,054 participants aged 16 to 99 from 237 countries, found that men reported higher loneliness than women. Furthermore, when examining studies that employ the UCLA loneliness scale, gender differences are often small or nonsignificant, and in those that do find differences, men often report higher loneliness [113]. With respect to nomophobia, findings are mixed, but several studies indicate that women are more likely than men to exhibit severe nomophobic tendencies [114]. This may reflect women’s greater discomfort or anxiety when unable to use their phone [115] or to communicate with others [116].

The most important finding of the present study is the confirmation of Hypothesis H6, which broadens our understanding of self-esteem as a mediating factor [117] and confirms its mediating role in the relationship between loneliness and phubbing. Previous studies offer similar insights. For example, Yang et al. [118], examining the relationship between parental phubbing and life satisfaction—including the mediating role of self-esteem and the moderating role of perceived social support—found that self-esteem partially mediated the relationship. Likewise, Wang and Qiao [119], in a study of over 2400 Chinese adolescents, examined bidirectional relationships among parental phubbing, self-esteem, and suicidal ideation and confirmed that self-esteem mediated the relationship between parental phubbing and suicidal ideation. Bitar et al. [74] found that self-esteem partially mediated the association between depressive temperament and phubbing, whereas emotional intelligence did not show a mediating effect.

Our findings extend this body of research by demonstrating that loneliness may lead to lower self-esteem, which in turn is associated with greater engagement in phubbing behaviors. Individuals experiencing loneliness may have a reduced sense of self-worth, which may prompt them to engage in phubbing more frequently, either as a coping strategy or to manage discomfort in face-to-face interactions by seeking connection in the virtual world. Although the correlational nature of the data prevents causal conclusions, the results highlight the importance of considering bidirectional dynamics and feedback loops. According to social cognitive theory, individuals with low self-esteem may engage in cognitive and behavioral processes that hinder social relationships and intensify loneliness [50]. For example, they may reach for their phone to avoid awkward or anxiety-provoking face-to-face interactions, further reinforcing loneliness. Recognizing these reciprocal dynamics is crucial for understanding the mediating role of self-esteem.

Human beings are inherently social, and satisfying relationships are essential for mental and physical health [120,121]. Thus, it is not surprising that loneliness has become a global concern, a public health issue [46], and a psychological challenge [122]. Loneliness is considered an indicator of social well-being [46] and affects overall quality of life [123,124]. The documented link between loneliness and phubbing enriches our understanding of both loneliness and the increasingly widespread phenomenon of phubbing [96], which is associated with smartphone use. Phubbing—disregarding others during social interactions—appears to have both intrapersonal and interpersonal negative consequences, such as harming social relationships through ignoring or excluding others [125,126], impairing communication between partners [127], and decreasing well-being while increasing feelings of ostracism among interaction partners [125,126,128].

In this context, the role of self-esteem as a mediator becomes particularly important, confirming that loneliness may reduce self-esteem, which in turn may contribute to phubbing behaviors. High self-esteem has beneficial effects across multiple areas of life, including forming satisfying relationships and experiencing greater well-being. However, some researchers also highlight the possibility of reverse causation, where life outcomes lead to changes in self-esteem [129], suggesting that self-esteem and life outcomes may exert mutual causal effects, forming a feedback loop [130]. Thus, a deeper analysis of self-esteem and its underlying mechanisms is crucial for both researchers and mental health professionals.

## 5. Limitations, Further Research, and Implications

One of the main limitations of the present study is that, although the mediation model identifies a potential explanatory mechanism (self-esteem), it does not establish causal relationships between loneliness and phubbing. The use of a cross-sectional design prevents the determination of cause-and-effect pathways, making it impossible to clearly identify which variable precedes the other. Nevertheless, the identified associations provide a valuable starting point for more detailed future research. Employing longitudinal designs in subsequent studies would allow for a more precise understanding of these relationships.

Another limitation concerns the use of an online survey, which does not fully capture the contextual factors associated with experiencing loneliness. Variables such as the quality of relationships with parents or friends were not considered. Including such variables in future studies could help clarify whether loneliness arises despite being in the presence of others or results from objective social isolation. Similarly, educational level was not included as a variable, and its consideration in future research would provide additional insight. Although the sample included individuals across early, middle, and late adulthood, it was not representative, which limits the generalizability of the findings. Future studies should prioritize recruiting underrepresented groups.

Furthermore, because the study relied on online convenience sampling rather than random sampling, the sample is unlikely to be fully representative of the general population, further limiting external validity. Future research would benefit from employing more diverse sampling strategies and, in particular, engaging a greater number of older adults to ensure broader population coverage.

Despite these limitations, the study makes a meaningful contribution to understanding the psychosocial factors associated with phubbing behaviors, as well as the mediating role of self-esteem—one of the fundamental psychological variables—in the relationship between loneliness and phubbing. Future research should focus both on a more detailed analysis of other psychosocial consequences of loneliness and on identifying additional factors that may contribute to phubbing behaviors. Moreover, we acknowledge that the study does not exhaust the range of possible mediators involved in this relationship. The inclusion of self-esteem offers an important extension of knowledge concerning this construct, whose direction of influence continues to be explored by researchers. Nonetheless, future studies should aim to identify additional mediators that may be significant in explaining how loneliness relates to phubbing.

The research conducted and the results obtained highlight two increasingly common phenomena—loneliness and phubbing—as well as self-esteem as a fundamental psychological variable. Understanding the relationships among these variables fills an important gap in the literature and contributes to a deeper understanding of the mechanisms underlying these experiences. Moreover, analyzing these variables may be valuable for raising awareness about technological hygiene.

## 6. Conclusions

Phubbing is a relatively new phenomenon, yet its widespread occurrence and negative impact on social relationships and mental health make it an important area of study. Phubbing can reduce relationship quality and satisfaction [14], intensify smartphone addiction [131], and even contribute to poorer academic performance and increased procrastination [132,133]. Recent research has begun to explore potential strategies for reducing phubbing [23,134,135], further emphasizing the relevance of this topic. Moreover, identifying the role of self-esteem—both in relation to loneliness and phubbing—extends existing findings and highlights the importance of self-esteem as a key mediating variable.

## Figures and Tables

**Table 1:** Sociodemographic characteristics of the study sample (*N* = 201).

Variables	*M*	*SD*	MIN	MAX	Skewness	Kurtosis
Loneliness	35.736	10.657	20.00	77.00	1.101	1.119
Intimate others	18.776	5.953	10	40	0.886	0.636
Social others	7.706	3.120	5	20	1.528	2.205
Belonging and affiliation	10.532	3.469	6	23	0.938	0.790
Phubbing	39.865	14.653	15	105	0.981	2.377
Nomophobia	13.094	5.559	4	28	0.545	−0.101
Interpersonal conflict	8.014	4.645	4	28	1.551	2.869
Self-isolation	8.293	4.352	4	28	1.591	3.474
Problem acknowledgment	10.462	4.385	3	21	0.094	−0.612
Self-esteem	29.442	6.866	10.00	40.00	−0.513	−0.280

**Table 2:** Values of Pearson’s r correlation coefficients between loneliness, phubbing, and self-esteem (*N* = 201).

Variables	LO	IO	SO	BA	PH	NO	IC	SI	PA	SE
Loneliness (LO)	1	0.920 ***	0.859 ***	0.847 ***	0.433 ***	0.202 **	0.401 ***	0.514 ***	0.254 ***	−0.450 ***
Intimate others (IO)		1	0.665 ***	0.610 ***	0.411 ***	0.241 **	0.348 ***	0.410 ***	0.290 ***	−0.414 ***
Social others (SO)			1	0.746 ***	0.390 ***	0.106	0.433 ***	0.526 ***	0.189 **	−0.424 ***
Belonging and affiliation (BA)				1	0.330 ***	0.129 ^t^	0.311 ***	0.482 ***	0.131 ^t^	−0.349 ***
Phubbing (PH)					1	0.795 ***	0.777 ***	0.768 ***	0.749 ***	−0.414 ***
Nomophobia (NO)						1	0.424 ***	0.447 ***	0.495 ***	−0.276 ***
Interpersonal conflict (IC)							1	0.561 ***	0.442 ***	−0.309 ***
Self-isolation (SI)								1	0.411 ***	−0.439 ***
Problem acknowledgment (PA)									1	−0.271 ***
Self-esteem (SE)										1

*** *p* < 0.001; ** p < 0.01; *p* > 0.05 ^t^; *p* < 0.1.

**Table 3:** Mean values, standard deviations, and *p*-values for differences between emerging adults and older adults in loneliness/its dimensions, phubbing/its dimensions, and self-esteem (*N* = 201).

Variables	Sex	*M*	*SD*	*p*
Loneliness (LO)	Emerging adults (EA)	37.157	11.666	0.013
	Older adults (OA)	33.587	8.545	
Intimate others (IO)	EA	19.776	6.560	0.002
	OA	17.262	4.527	
Social others (SO)	EA	8.090	3.237	0.031
	OA	7.125	2.856	
Belonging and affiliation (BA)	EA	10.611	3.638	0.692
	OA	10.412	3.216	
Phubbing (PH)	EA	43.785	14.614	0.001
	OA	33.937	12.652	
Nomophobia (NO)	EA	14.405	5.565	0.001
	OA	11.112	4.958	
Interpersonal conflict (IC)	EA	8.537	4.813	0.050
	OA	7.225	4.290	
Self-isolation (SI)	EA	8.983	4.566	0.005
	OA	7.250	3.803	
Problem acknowledgment (PA)	EA	11.859	4.064	0.001
	OA	8.350	4.009	
Self-esteem (SE)	EA	27.942	7.072	0.001
	OA	31.712	5.891	

**Table 4:** Mean values, standard deviations, and *p*-values for differences between women and men in loneliness/its dimensions, phubbing/its dimensions, and self-esteem (*N* = 201).

Variables	Sex	*M*	*SD*	*p*
Loneliness (LO)	Women (W)	34.977	10.574	0.168
	Men (M)	37.157	10.741	
Intimate others (IO)	W	18.610	6.111	0.591
	M	19.085	5.676	
Social others (SO)	W	7.450	2.882	0.134
	M	8.185	3.494	
Belonging and affiliation (BA)	W	10.160	3.499	0.037
	M	11.228	3.328	
Phubbing (PH)	W	40.847	14.563	0.195
	M	38.028	14.751	
Nomophobia (NO)	W	13.931	5.672	0.003
	M	11.528	5.015	
Interpersonal conflict (IC)	W	7.923	4.571	0.704
	M	8.185	4.810	
Self-isolation (SI)	W	8.167	4.293	0.577
	M	8.528	4.484	
Problem acknowledgment (PA)	W	10.824	4.407	0.110
	M	9.785	4.293	
Self-esteem (SE)	W	29.122	6.971	0.366
	M	30.042	6.673	

**Table 5:** Role of self-esteem in the relationship between loneliness and phubbing (*N* = 201).

Model	a Path	b Path	c Path	c’ Path	Indirect Effect	B (SE)	Lower CI	Upper CI
LO → S-E → PHUB	−0.289 ***	−0.587 ***	0.594 ***	0.424 ***	0.170	0.051	0.078	0.275
IO → S-E → PHUB	−0.477 ***	−0.628 ***	1.010 ***	0.710 ***	0.300	0.091	0.137	0.501
SO → S-E → PHUB	−0.933 ***	−0.647 ***	1.831 ***	1.227 ***	0.604	0.173	0.294	0.969
BA → S-E → PHUB	−0.689 ***	−0.726 ***	1.392 ***	0.890 **	0.501	0.152	0.219	0.816
LO → S-E → NO	−0.289 ***	−0.187 **	0.105 **	0.051 (ns)	0.054	0.020	0.015	0.094
IO → S-E → NO	−0.477 ***	−0.171 **	0.225 ***	0.143 *	0.082	0.033	0.020	0.151
SO → S-E → NO	−0.933 ***	−0.227 ***	0.188 (ns)	−0.024 (ns)	0.212	0.066	0.087	0.348
BA → S-E → NO	−0.689 ***	−0.212 ***	0.206 (ns)	0.060 (ns)	0.146	0.053	0.053	0.261
LO → S-E → IC	−0.289 ***	−0.109 *	0.174 ***	0.143 ***	0.031	0.016	0.001	0.064
IO → S-E → IC	−0.477 ***	−0.135 **	0.271 ***	0.207 ***	0.064	0.028	0.013	0.124
SO → S-E → IC	−0.933 ***	−0.103 *	0.644 ***	0.547 ***	0.096	0.052	−0.006	0.202
BA → S-E → IC	−0.689 ***	−0.154 **	0.416 ***	0.309 **	0.106	0.044	0.026	0.198
LO → S-E → SI	−0.289 ***	−0.165 ***	0.209 ***	0.162 ***	0.047	0.014	0.021	0.078
IO → S-E → SI	−0.477 ***	−0.205 ***	0.299 ***	0.201 ***	0.098	0.029	0.048	0.166
SO → S-E → SI	−0.933 ***	−0.166 ***	0.733 ***	0.578 ***	0.155	0.047	0.070	0.260
BA → S-E → SI	−0.689 ***	−0.195 ***	0.604 ***	0.469 ***	0.134	0.042	0.061	0.228
LO → S-E → PA	−0.289 ***	−0.125 *	0.104 ***	0.068 *	0.036	0.014	0.010	0.068
IO → S-E → PA	−0.477 ***	−0.116 *	0.213 ***	0.158 **	0.055	0.023	0.014	0.108
SO → S-E → PA	−0.933 ***	−0.148 **	0.265 ***	0.126 (ns)	0.138	0.049	0.051	0.245
BA → S-E → PA	−0.689 ***	−0.163 ***	0.165 (ns)	0.052 (ns)	0.113	0.042	0.041	0.207

*** *p* < 0.001; ** *p* < 0.01; * *p* < 0.05; ns—not significant; LO—Loneliness; IO—Intimate others; SO—Social others; BA—Belonging and affiliation; S-E—Self-esteem; PHUB—Phubbing; NO—Nomophobia; IC—Interpersonal conflict; SI—Self-isolation; PA—Problem acknowledgment.

## Data Availability

Data supporting reported results can be found at https://osf.io/6vmkj/overview?view_only=dd24766ea14a42aabb346885074b96b2 (accessed on 19 October 2025).
